# Utilization of patient-reported outcomes to assess adherence to relugolix when combined with stereotactic body radiation therapy for intermediate to high-risk prostate cancer

**DOI:** 10.3389/fonc.2025.1540482

**Published:** 2025-01-30

**Authors:** Kelly Gaudian, Min Jung Koh, Min Ji Koh, Ryan R. Collins, Shaine Eden, Zoya Zwart, Malika Danner, Alan Zwart, Mark Fallick, Deepak Kumar, Paul Leger, Nancy A. Dawson, Simeng Suy, Sean P. Collins

**Affiliations:** ^1^ Washington University School of Medicine in St. Louis, St. Louis, MO, United States; ^2^ School of Medicine, Georgetown University, Washington, DC, United States; ^3^ College of William and Mary, Williamsburg, VA, United States; ^4^ Systems Medicine Program, Georgetown University Medical Center, Washington, DC, United States; ^5^ Department of Oncology, Lombardi Comprehensive Cancer Center, Georgetown University Medical Center, Washington, DC, United States; ^6^ Department of Radiation Oncology, University of South Florida (USF) Health Morsani College of Medicine, Tampa, FL, United States; ^7^ Novartis, US Medical Affairs, Oncology, East Hanover, NJ, United States; ^8^ Biotechnology Research Institute, North Carolina Central University, Durham, NC, United States

**Keywords:** SBRT (stereotactic body radiation therapy), relugolix, ADT (androgen deprivation therapy), prostate cancer, medication adherence

## Abstract

**Introduction:**

Injectable GnRH receptor agonists have been shown to improve cancer control when combined with radiotherapy (RT). Relugolix is an oral GnRH receptor antagonist that achieves rapid testosterone suppression. Non-adherence to oral medications due to poor social support or bothersome side effects may increase the risk of cancer recurrence. This prospective study sought to evaluate early testosterone suppression and relugolix drug adherence when combined with prostate stereotactic body radiation therapy (SBRT). Utilization of patient-reported outcomes (PROs) to assess oral medication adherence and guide intervention may improve the appropriate utilization of oral medications. This study focuses on the use of the Simplified Medication Adherence Questionnaire (SMAQ) as a tool to assess relugolix adherence.

**Methods:**

Relugolix was initiated at least 2 months prior to questionnaire administration. Adherence was assessed using the SMAQ. Total testosterone levels were obtained at the time of SMAQ administration. Castration was defined as serum testosterone ≤ 50 ng/dL. Poor drug adherence was delineated as failure to reach castration or non-adherence per the SMAQ (any non-adherence answer, missed > 2 doses in last week or since last visit). To compare the demographic and clinical characteristics of patients who adhered to treatment versus who did not, t-test, Wilcoxon rank sum test, Chi-square test, and Fisher’s exact test were used. A p-value < 0.05 determined statistical significance.

**Results:**

Between August 2021 and December 2023, 78 men were treated at Georgetown with relugolix and prostate SBRT per an institutional protocol. The median age was 72, and 41% of patients were non-white. Patients initiated relugolix at a median of 4 months prior to the SMAQ (2-19 months). 96% of patients achieved castration (≤ 50 ng/dL) at the time of the SMAQ. 96% of men reported always taking relugolix at the appropriate time. 1% discontinued medication due to bothersome side effects, 17% reported forgetting to take the medication, and 4% reported missing a dose during the weekend. 98% and 93% did not miss a dose more than 2 times in the last week and since the last visit, respectively. Overall patient-reported drug adherence was 75%. No patient demographic or clinical characteristic predicted non-adherence.

**Discussion:**

Relugolix allows for high rates of castration and drug adherence when combined with prostate SBRT. Monitoring drug adherence during treatment allows for prompt detection of non-adherence and timely intervention. Future studies should focus on how to optimally incorporate this questionnaire into patient management.

## Introduction

Injectable GnRH receptor agonists have been shown to improve cancer control when combined with radiotherapy (RT) (1). Androgen deprivation therapy (ADT) in conjunction with conventionally fractionated radiation therapy significantly improves metastases-free and overall survival in men with prostate cancer (2). As with external beam radiation therapy (EBRT), early data suggests that the addition of ADT to SBRT for unfavorable prostate cancer may also reduce local persistence of disease and biochemical recurrence (3, 4). Unfortunately, ADT combined with RT remains underutilized, possibly due to bothersome persistent side effects and its potentially negative impact on cardiovascular comorbidities (5).

Relugolix is an oral ADT that suppresses gonadotropic release from the pituitary gland, thus decreasing concentrations of testosterone (6). The HERO study investigated the efficacy of this oral GnRH receptor antagonist compared to GnRH agonist leuprolide. The randomized Phase 3 trial demonstrated the superiority of relugolix in achieving and maintaining castration (7). Men treated with relugolix maintained castration through 48 weeks at a rate of 96.7% compared to 88.8% with leuprolide (7). The study exhibited a 99% adherence rate with oral relugolix, although most patients had daily audible reminders to take their pill (7). Similarly, neoadjuvant/adjuvant relugolix (6 months) has been studied in unfavorable localized prostate cancer in combination with conventionally fractionated radiation therapy (79.2 Gy in 44 fractions) (8). With relugolix, 95% achieved castration (total testosterone < 50 ng/dL, 1.73 nmol/L) and 82% reached profound castration (total testosterone < 20 ng/dL; 0.7 nmol/L). As with the HERO study, interventions to improve adherence were utilized (8).

While oral ADTs have potential advantages, their real-world effectiveness is dependent on patient adherence (9). Non-adherence to ADT could lead to increased rates of biochemical recurrence and prostate cancer-specific death. Adherence is defined as taking a medication exactly as prescribed. Generally, an adherence rate of > 80% is required for optimal therapeutic efficacy. Unfortunately, medication non-adherence is common, with up to 50% of patients self-reporting non-adherence (10). The estimated rate of non-adherence in prostate cancer patients receiving oral anti-androgens for metastatic disease is 5-10% (11–14). Factors associated with non-adherence include advanced age, multiple daily doses, side-effect severity, length of use, and inadequate patient education (15). Bothersome side effects such as hot flashes, fatigue, and decreased libido may lead to drug holidays and/or early cessation (16). This may be a bigger problem in minority and other underserved populations (15, 16).

Currently, a test for relugolix serum levels is not clinically available. However, adherence can be measured using indirect methods (i.e., serum testosterone levels) or subjective measures, such as patient-reported outcomes (PROs). PROs allow for early assessment that is both affordable and non-invasive. The Simplified Medication Adherence Questionnaire (SMAQ) is a commonly used psychometrically validated questionnaire with six questions to quantify omissions and barriers to adherence (forgetfulness, carelessness, skipping doses due to adverse side effects) (17, 18). This information can be utilized to develop individualized approaches tailored to a patient’s specific reasons for and level of non-adherence.

Adherence to medications assessed by SMAQ has been studied for a variety of medications utilized in different disease states, such as chronic myeloid leukemia (CML), HIV, bipolar disorder, acute coronary syndrome (ACS), Parkinson’s disease, and more (18–25). These studies have identified numerous predictive factors of SMAQ-reported adherence, including patient education level, employment status, cognitive functioning, alcohol consumption, and negative attitude toward treatment (19–21). However, other studies utilizing the SMAQ have found no correlation between compliance and social demographic data (23, 25). Additionally, adherence to therapy has been shown to be affected by treatment-related toxicities and patient quality of life (22).

Limited data are available on patient adherence to oral ADT in men receiving radiation therapy for localized prostate cancer. Further knowledge in this area would aid in developing approaches to improve adherence. The objective of this study was to prospectively collect and report patient-reported relugolix adherence at the start of prostate SBRT for intermediate to high-risk prostate cancer. In addition, we demonstrate the feasibility of incorporating the SMAQ into daily clinical practice during a course of RT and its utility in improving drug adherence.

## Materials and methods

We conducted an IRB-approved, prospective study (IRB 12-1175) of men with prostate cancer treated with short-term relugolix and prostate SBRT at Georgetown University Hospital. Patient and treatment characteristics, such as age, race, partner status, comorbidities, BMI, and risk group, were acquired from the medical records.

### Drug treatment

Relugolix was initiated with a loading dose of 360 mg on the first day and continued treatment with a 120 mg dose taken orally once daily at approximately the same time each day. If a dose was missed, and it was 12 hours or less since they were supposed to take their pill, patients were instructed to take the pill as soon as they remembered. If the dose was missed by more than 12 hours, patients were instructed not to take the missed dose and to take their next dose at the regular time the next day. Patients were informed that they would take the pills for approximately three to six months and at the end of this period, they would discontinue the medication, with expected rapid testosterone recovery and side effect resolution (7). Relugolix treatment duration varied within the patient cohort due to heterogeneity in the timing of initiating prostate SBRT. SBRT was delayed due to logistical challenges, including patient availability, fiducial placement scheduling, and treatment planning MRI availability.

### SBRT treatment planning and delivery

Prostate SBRT was delivered using the CyberKnife robotic radiosurgical system (Accuray Inc., Sunnyvale, CA) as previously described (26, 27). Approximately two months following the initiation of relugolix, gold fiducials were transperineally placed into the prostate. One week after fiducial placement, CT and high-resolution MR images were obtained for treatment planning. In general, patients initiated prostate SBRT 2-4 weeks following treatment simulation. Radiation was delivered every other day in 5 approximately 35-minute treatments.

### Follow-up and assessment

PRO assessments were prospectively collected at the first doctor visit after the initiation of relugolix. Total testosterone levels were collected at the time of questionnaire administration. We defined both effective and profound castration as serum testosterone of <50 ng/dL (¾1.73 nmol/L) and ¾ 20 ng/dL (¾0.7 nmol/L), respectively, as previously described (7). The SMAQ is a validated tool that measures drug adherence (18). A patient is considered non-compliant via the SMAQ if he responds to any question with a non-adherent answer or has missed two or more doses in the last week or two consecutive days since the last healthcare visit. Patients were grouped into adherent and non-adherent populations based on these criteria. To compare the demographic and clinical characteristics of patients who adhered to treatment versus those who did not, t-test, Wilcoxon rank sum test, Chi-square test, and Fisher’s exact test were used. A p-value < 0.05 determined statistical significance.

## Results

### Patient, treatment, and tumor characteristics

Between August 2021 and December 2023, 78 men were treated at Georgetown with relugolix and prostate SBRT per an institutional protocol. The median age was 72, 41% of patients were non-white, 22% were un-partnered, and 12% had a moderate to severe comorbidity index (**Table 1**). Patients initiated relugolix at a median of 4 months prior to the SMAQ (range of 2-19 months).

**Table 1:** Demographic and clinical characteristics.

Characteristics	No. (%)			P value
	All	Adherent	Not Adherent	
	(n = 78)	(n = 58)	(n = 20)	
Age (y), Median (IQR)	72 (68-77)	71 (67-77)	75 (68-79)	0.5^a^, 0.9^b^
<60	5 (6)	4 (7)	1 (5)	
60-69	23 (30)	17 (29)	6 (30)	
70-79	34 (44)	26 (45)	8 (40)	
>80	16 (21)	11 (19)	5 (25)	
Race				0.1^a^, 0.2^b^
White	46 (59)	37 (64)	9 (45)	
Black	25 (32)	15 (26)	10 (50)	
Other	7 (9)	6 (10)	1 (5)	
Gleason Score				0.6^a^, 0.6^b^
3 + 3 = 6	3 (4)	3 (5)	0 (0)	
3 + 4 = 7	17 (22)	13 (22)	4 (20)	
4 + 3 = 7	40 (51)	31 (53)	9 (45)	
4 + 4 = 8	15 (19)	9 (16)	6 (30)	
4 + 5 = 9	3 (4)	2 (3)	1 (5)	
Risk Group				0.5^a^, 0.6^b^
Low	1 (1)	1 (2)	0 (0)	
Intermediate	57 (73)	44 (76)	13 (65)	
High	19 (24)	12 (21)	7 (35)	
Recurrent	1 (1)	1 (2)	0 (0)	
Prostate Volume (cc), Median (IQR)	38 (26-50)	39 (26-52)	37 (28-47)	0.6^a^
Achieved Castration (Testosterone <50ng/dL)				1.0^a^, 1.0^b^
Yes	75 (96)	56 (97)	19 (95)	
No	3 (4)	2 (3)	1 (5)	
Partner Status				1.0^a^, 1.0^b^
Partnered	42 (78)	30 (77)	12 (80)	
Single	12 (22)	9 (23)	3 (20)	
Comorbidity Index (CCI)				0.9^a^, 1.0^b^
0: None	31 (61)	22 (60)	9 (64)	
1-2: Mild	14 (28)	10 (27)	4 (29)	
3-4: Moderate	4 (8)	3 (8)	1 (7)	
=>5: Severe	2 (4)	2 (5)	0 (0)	

a P values for the comparison between patients who were adherent to relugolix versus non-adherent were calculated using t-test, Wilcoxon rank sum test, and chi-square test for normally distributed continuous, non-normally distributed continuous, and categorical variables, respectively.

b P-values based on Fisher’s exact test due to some small cell counts.

### Total testosterone levels

See **Table 2** for a summary of testosterone responses following relugolix. At the time of questionnaire administration, 96% of patients achieved a testosterone level ≤50 ng/dl (effective castration) while 90% reached testosterone ≤20 ng/dl (profound castration).

**Table 2:** Testosterone levels.

Characteristics	No. (%)			P value
	All	Adherent	Not Adherent	
	(n = 78)	(n = 58)	(n = 20)	
Achieved castration (Testosterone <50ng/dL)				
Yes	75 (96)	56 (97)	19 (95)	1.0^a^, 1.0^b^
No	3 (4)	2 (3)	1 (5)	
Achieved profound castration (Testosterone <20ng/dL)				
Yes	70 (90)	53 (91)	17 (85)	0.7^a^, 0.4^b^
No	8 (10)	5 (9)	3 (15)	

a P values for the comparison between patients who were adherent to relugolix versus non-adherent were calculated using t-test, Wilcoxon rank sum test, and chi-square test for normally distributed continuous, non-normally distributed continuous, and categorical variables, respectively.

b P-values based on Fisher’s exact test due to some small cell counts.

### Patient-reported adherence

96% of patients achieved castration (≤ 50 ng/dL) at the time of the SMAQ. 96% of men reported always taking relugolix at the appropriate time. 1% discontinued medication due to bothersome side effects, 17% reported forgetting to take the medication, and 4% reported missing a dose during the weekend. 98% and 93% did not miss a dose more than 2 times in the last week and since the last visit, respectively. Overall patient-reported drug adherence was 75% (**Table 3**). No patient demographic or clinical characteristic predicted non-adherence. 

**Table 3:** Simplified Medication Adherence Questionnaire (SMAQ) results.

Characteristics	No. (%)
Always Taken at Appropriate Time	
Yes	75 (96)
No	3 (4)
Discontinued Medication Due to Bothersome Side Effects	
Yes	1 (1)
No	77 (99)
Forgotten to Take the Medication	
Yes	13 (17)
No	65 (83)
Forgotten to Take the Medication During the Weekend	
Yes	3 (4)
No	75 (96)
# of Missed Doses in the Last Week	
Never	70 (90)
1-2 times	6 (8)
3-5 times	1 (1)
6-7 times	1 (1)
# of Missed Doses Since Last Visit (Days)	
0	61 (78)
1	5 (6)
2	6 (8)
>3	5 (6)
NA	1 (1)

## Discussion

This study utilizes a regimen combining an oral GnRH receptor antagonist, relugolix, and SBRT for localized prostate cancer. Hypofractionated and ultrahypofractionated radiotherapy (SBRT) has been shown to be an effective curative treatment option for patients with localized prostate cancer when compared to conventionally fractioned radiation therapy (CFRT), with favorable biochemical control and toxicity levels (28, 29). ADT can be utilized as a complementary treatment to SBRT, as studies have shown that a combination of the two modalities can provide improved outcomes (3, 4).

Drug adherence is an important consideration when utilizing oral androgen deprivation therapies in combination with prostate SBRT. Measuring serum testosterone levels is a commonly employed indirect measure to assess drug adherence (30). However, interval testosterone levels may not capture short-term drug interruptions that could negatively impact cancer control. A better understanding of patient adherence patterns would aid in designing approaches to improve it.

Prior studies of relugolix utilized daily reminders which likely improved compliance rates and ensured continued testosterone suppression (7, 8). Due to administrative burden, we did not utilize scheduled cues as part of our study. Nonetheless, our self-reported adherence (SMAQ) was medium (60-80%) and higher than previous studies utilizing the SMAQ questionnaire (**Table 4**; 38-64%). Over 95% of patients reported taking the drug at the same time every day. It was reassuring that only 3% of patients skipped the medication due to bothersome side effects. The main reason for non-adherence was forgetfulness. However, the 20% of men who reported forgetting to take the medication is relatively low for an elderly male patient population (31). The results are reassuring, considering greater than a third of our patients were minorities, 22% were un-partnered and 12% had a moderate to severe comorbidity index (**Table 1**).

**Table 4:** Medication adherence assessed by Simplified Medication Adherence Questionnaire (SMAQ) in varying disease states.

Disease	Drug	Patients (n)	Adherence Score
Chronic myeloid leukemia (CML)	Tyrosine kinase inhibitors (TKIs) Imatinib (54.8%), nilotinib (63.6%), dasatinib (54.3%)	130	56.9% (19)
HIV	Antiretroviral therapy (ART); NNRTI (nonnucleoside reverse transcriptase inhibitor), PI (Protease inhibitors)	150	64.3% (20)
Bipolar disorder	Antipsychotics, anticonvulsants, lithium	578	39.9% at baseline (21)
HIV	RPV/FTC/TDF	125	54.4% at baseline (22)
Acute coronary syndrome (ACS)		100	38% at 12 months post-hospitalization (23)
Parkinson’s disease	Levodopa; Dopamine agonists; MAO-B inhibitors	800	41.75% (24)
Hypertensive disorders of pregnancy, fetal growth restriction (FGR), intrauterine fetal death	Aspirin	53	53.7% (25)
Renal transplant	Tacrolimus	144	61% (18)

Limitations of our investigation are secondary to its small size and variations in treatment scheduling. Although we aimed to treat all patients with relugolix for a total of 4-6 months with initiation 2 months prior to SBRT, there was heterogeneity in the timing of initiating prostate SBRT. Due to the small sample size, we did not examine whether there were differences in outcomes depending on the timeliness of SBRT. We employed a short course of administration, and adherence may not be as high with a long course (21, 24). Additionally, the SMAQ is not validated for hormonal agents, and the SMAQ did not assess medication concerns associated with non-adherence (i.e., necessity, harmfulness, etc.). Due to high levels of castration, we could not definitively correlate results with an objective measure of adherence.

## Conclusion

Relugolix allows for high rates of castration and drug adherence when combined with SBRT. Monitoring drug adherence during treatment may allow for prompt detection of non-adherence and timely intervention. Future studies should focus on how to optimally incorporate the SMAQ into patient management.

## Data Availability

The raw data supporting the conclusions of this article will be made available by the authors, without undue reservation.
